# Cardiac Tamponade in Pulmonary Hypertension: Management Using Right Heart Catheterization Guidance

**DOI:** 10.1016/j.jscai.2025.102619

**Published:** 2025-03-07

**Authors:** Abraham Shin, Jean-Claude Asaker, J. Dawn Abbott, Christopher J. Mullin, Saraschandra Vallabhajosyula

**Affiliations:** a Department of Medicine, Warren Alpert Medical School of Brown University, Providence, Rhode Island; b Division of Cardiology, Department of Medicine, Warren Alpert Medical School of Brown University, Providence, Rhode Island; c Brown University Health Cardiovascular Institute, Providence, Rhode Island; d Division of Pulmonary, Critical Care, and Sleep Medicine, Department of Medicine, Warren Alpert Medical School of Brown University, Providence, Rhode Island

**Keywords:** cardiac tamponade, invasive hemodynamics, pericardial effusion, pulmonary hypertension, right heart catheterization

Pericardial effusion portends a poor prognosis in pulmonary hypertension (PH) and is indicative of chronically increased right atrial pressure. Cardiac tamponade can be both difficult to diagnose and manage in PH, as abrupt reduction in pericardial pressure can precipitate worsening right ventricular failure from decreased right ventricular preload and worsening right ventricular dilation in setting of high afterload. This report describes the management of tamponade in a patient with known PH using continuous invasive pressure monitoring.

A 63-year-old man with muscular dystrophy, restrictive lung disease, and group 3 PH (diagnosed previously by right heart catheterization; previous 3 L of oxygen at baseline) with chronic right ventricular dysfunction presented with worsening dyspnea. His therapy on admission included nocturnal noninvasive ventilation, diuretics, tadalafil, and ambrisentan. He was hypotensive (87/53 mm Hg), and hypoxemic (12-L oxygen nonrebreather mask) and had evidence of hypoperfusion (lactic acidosis and acute liver and kidney injury). Chest x-ray revealed bilateral pleural effusions with pulmonary edema (Figure 1A). Transthoracic echocardiogram demonstrated a new circumferential effusion with reduced left ventricular filling, right ventricular dysfunction without collapse, plethoric inferior vena cava, and no respiratory variations in mitral (21%) and tricuspid (20%) inflow velocities (Figure 1B, C, Supplementary Figures S1 and S2, and Supplementary Videos 1 and 2).

**Figure 1:**
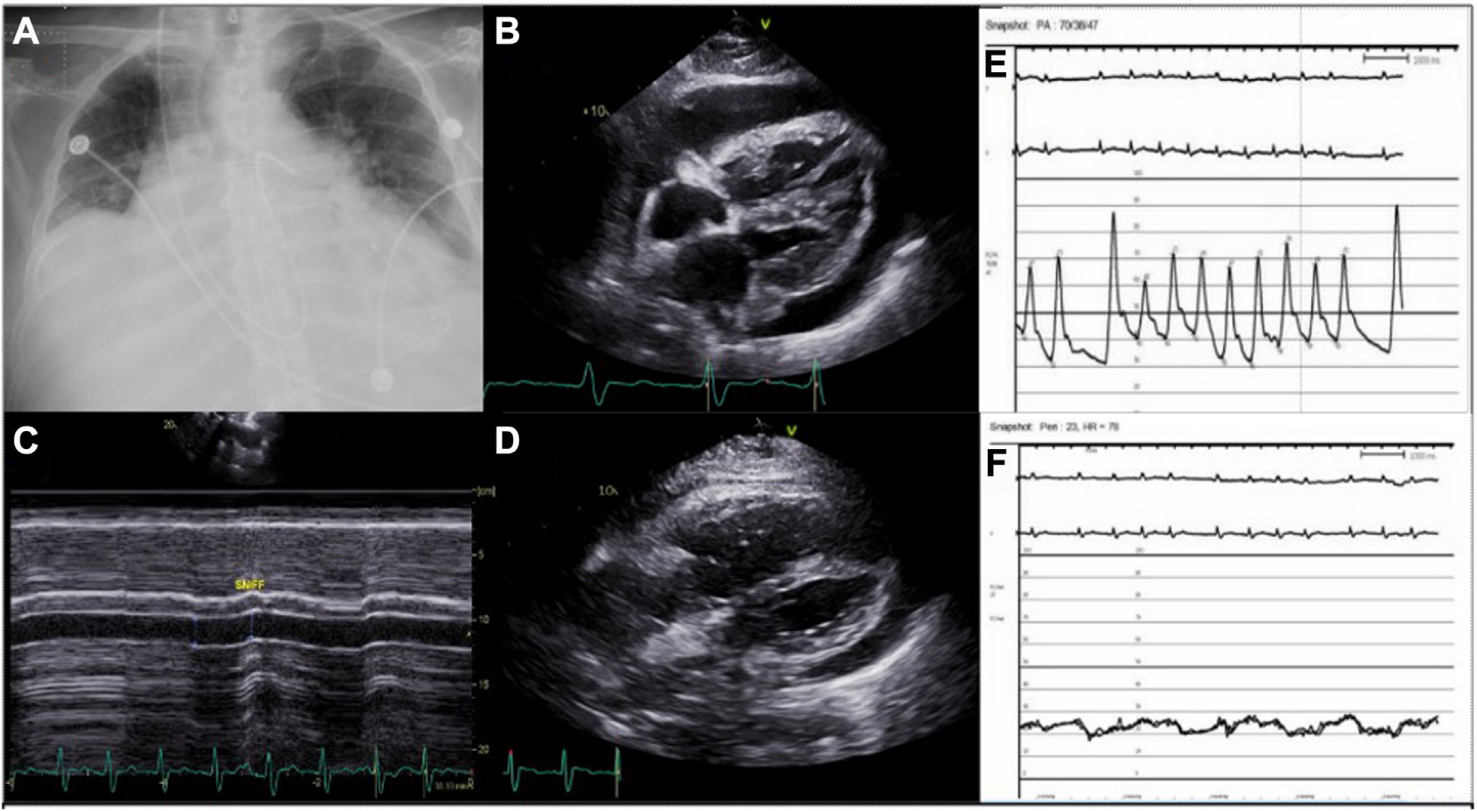
**Management of cardiac tamponade in pulmonary hypertension.** (A) Chest x-ray demonstrating bilateral pleural effusions with pulmonary edema and right heart catheter in situ. (B) Predrainage transthoracic echocardiogram (TTE) demonstrating large pericardial effusion with right ventricular (RV) collapse tamponade physiology (subcostal view). (C) Inferior vena cava of >21.0 mm without variation on sniff test. (D) Postconservative drainage TTE demonstrating interval decrease in the pericardial effusion with improvement on RV function. (E) Hemodynamic tracings initially with pulmonary artery pressure of 70/36 (47) mm Hg. (F) Opening pericardial pressure of 23 mm Hg.

Given the high suspicion of atypical cardiac tamponade, right heart catheterization was performed. It revealed near equalization of diastolic pressures: mean right atrium, 20 mm Hg; right ventricle 67/23 mm Hg; pulmonary artery, 70/38 (mean, 47) mm Hg; and pulmonary capillary wedge pressure, 25 mm Hg (Figure 1E). Reduced cardiac output (2.97 L/min) and cardiac index (1.63 L/min/m^2^) were consistent with obstructive shock. Subxiphoid pericardial access was established, and opening pericardial pressure was 23 mm Hg (Figure 1F). After every 100 mL removal, the surgeons waited 2 to 3 minutes and remeasured right atrial and pericardial pressures to assess the right ventricular filling. After removing 500 mL, right atrial and pericardial pressures decreased to 18 and 12 mm Hg, respectively, and systemic blood pressure (117/59 mm Hg) and cardiac index (2.82 L/min/m^2^) normalized. Intraprocedural echocardiogram showed a decreased pericardial effusion, improved biventricular filling, and improved right ventricular function (Figure 1D). The patient was admitted to the intensive care unit with a pericardial drain and Swan-Ganz catheter for close hemodynamic monitoring. Intravenous diuresis and serial low-volume pericardial drainage were performed to maintain right atrial pressure of 10 mm Hg and avoid worsening right ventricular failure.

The patient reported symptomatic improvement, and laboratory markers for end-organ damage from obstructive shock improved within a few days. Owing to concerns that the progression of PH led to pericardial effusion, selexipag was added and uptitrated over the subsequent weeks. Serial echocardiography over the subsequent months demonstrated resolution of pericardial effusion and improvement of PH.

Cardiac tamponade in PH is associated with increased all-cause mortality, and pericardiocentesis is further associated with higher in-hospital mortality and postprocedural shock.^1^^,^^2^ This case adds to established literature showing successful treatment of cardiac tamponade in PH with gradual pericardial drainage over several days to avoid worsening right ventricular failure.^3^^,^^4^ Despite its efficacy, these data need further validation in larger cohort studies.

## Figures and Tables

**Supplementary Figures:**
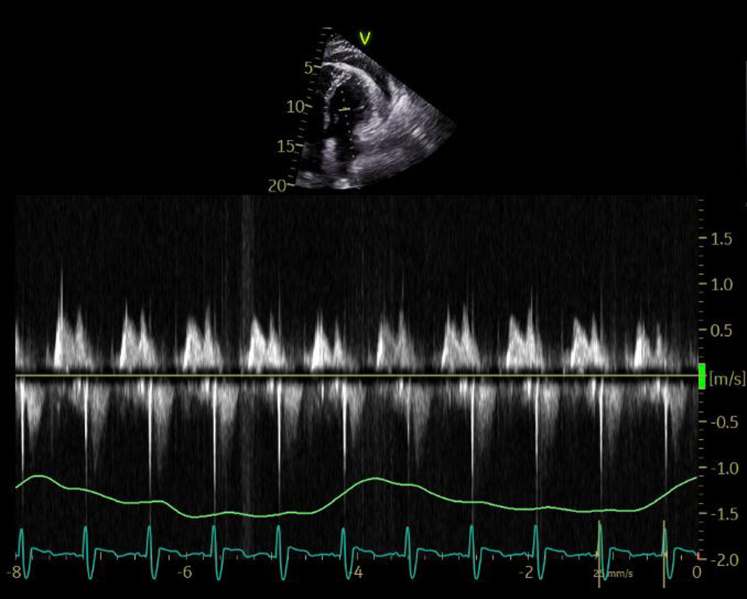

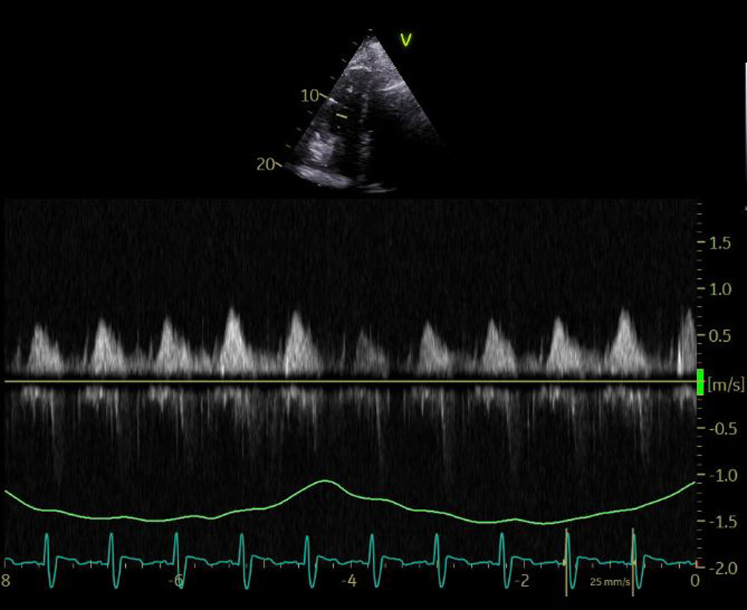
Mitral (21%) (Figure S1) and tricuspid (20%) (Figure S2) valve inflow Doppler velocities showing minimal respiratory variation

## Supplementary material

Supplemental Video 1 Large pericardial effusion with right ventricular collapse in a subcostal view.1

Supplemental Video 2 Apical 4-chamber view.2

## Footnotes

To access the supplementary material accompanying this article, visit the online version of the *Journal of the Society for Cardiovascular Angiography & Interventions* at 10.1016/j.jscai.2025.102619.
